# CARE DEPENDENCY: EXPERIENCES AND EXPECTATIONS OF COMMUNITY-DWELLING OLDER ADULTS

**DOI:** 10.1093/geroni/igz038.2326

**Published:** 2019-11-08

**Authors:** Jan S Jukema, Sharon Oude Veldhuis, Jacqueline Van Alphen, Jopie Jorritsma, Frits De Lange

**Affiliations:** 1 Saxion University of Applied Sciences, Deventer, Netherlands; 2 Saxion University of Applied Sciences, Zwolle, Netherlands; 3 Protestant Theological University, Groningen, Netherlands

## Abstract

“Not to be a burden” is a common phrase used by community-dwelling older adults in discussing their dependency on others in care for their daily life. This attitude may lead to conflicts with relatives, neighbors, or professionals when in their opinion, care is necessary and, ultimately, may result in unmet care needs. The goal of this study is to gain a better understanding of how older adults experience their increased dependency on others and to contribute to the development of an ethic of care. Thirty-two participants of a larger research sample (n=64) from a descriptive qualitative research were purposefully selected, resulting in an equal distribution of the following variables: gender, living situation, living with or without partner, and having children or not. From a multiphase qualitative analysis with five researchers, including two senior citizens four themes emerged: (1) relationships in the context of care; (2) experiences with giving, receiving and asking for care; (3) future perspectives towards receiving and asking for care; and (4) actual practices of caregiving and receiving. Our study clarifies how community-dwelling older adults deal with the changes in their dependency on others. The study results highlight particular dynamics which appear, at least, partly in contrast with current policy regarding care at home. Moreover, it contributes to an empirical refinement of the concepts of dependency and interdependency in an ethic of care. Further studies are needed to clarify the influential factors on asking for care in diverse groups of older adults and the response from their network.

